# PI3K Signaling in B Cell and T Cell Biology

**DOI:** 10.3389/fimmu.2014.00557

**Published:** 2014-11-03

**Authors:** Klaus Okkenhaug, Martin Turner, Michael R. Gold

**Affiliations:** ^1^Laboratory of Lymphocyte Signalling and Development, Babraham Institute, Cambridge, UK; ^2^Department of Microbiology and Immunology, University of British Columbia, Vancouver, BC, Canada

**Keywords:** PI3K/AKT/mTOR, PI3K pathway inhibitors, T cell, B cell, signal transduction

The drug Idelalisib is the first PI3K inhibitor to be approved by the FDA for clinical use. It is therefore timely to take stock of our current understanding of the role of PI3Ks in the immune system. The phosphoinositide 3-kinases (PI3Ks) control many key functions in immune cells (1). PI3Ks phosphorylate PtdIns(4,5)P_2_ to yield PtdIns(3,4,5)P_3_, which acts as a second messenger signaling molecule that controls the activation of Akt and other proteins with PH domains. Initially, PI3K inhibitors such as Wortmannin, LY294002, and Rapamycin were used to establish a central role for PI3K pathway in immune cells. More recent progress in understanding the role of this pathway in cells of the immune system has been made through the generation of wide range of gene-targeted mouse models as well as with the development of highly selective small molecule inhibitors, culminating in the FDA approval of the PI3Kδ inhibitor Idelalisib in 2014. Together, lab experiments, preclinical, and clinical trials have revealed how PI3Ks control B cell and T cell development, T helper cell differentiation, regulatory T cell (Treg) development and function, B cell and T cell trafficking, immunoglobulin class switching, and much more.

The class I PI3Ks are heterodimers composed of a catalytic subunit and a regulatory subunit. The p110α, p110β, and p110δ catalytic subunits form heterodimers with either of the p85α, p55α, p50α, p85β, or p55γ regulatory subunits, each of which contains SH2 domains that are engaged by tyrosine phosphorylated proteins. By contrast, the p110γ catalytic subunit forms heterodimers with either the p84 or p101 regulatory subunits that are recruited to the Gβγ subunits released by G-protein coupled receptors. The respective heterodimers are often referred to as PI3Kα, PI3Kβ, PI3Kδ, or PI3Kγ, respectively. Progress has been made in understanding the often non-redundant roles of each of these PI3K isoforms in immunity (2). The majority of these efforts have been directed at PI3Kδ and PI3Kγ, which are highly expressed in cells of the immune system.

mTOR inhibitors such as rapamycin are already established as among the most commonly used drugs to prevent transplant rejection and are increasingly also evaluated for the use in cancer (3). Most recently, the PI3Kδ-selective inhibitor Idelalisib (CAL-101) has received FDA approval for the treatment of chronic lymphocyte leukemia and indolent non-Hodgkin’s lymphoma following on from successful clinical trials showing dramatic improvements in progress-free survival (4, 5). Many other PI3K inhibitors are going through clinical trials with the aim to treat cancers, and inflammatory and autoimmune diseases.

This eBook contains 11 chapters that cover different roles of the PI3K and mTOR pathways, primarily in B cells and T cells. Limon and Fruman review the role of PI3Ks in B cell development and activation and also consider how the related Akt, Foxo, and mTOR pathways affect B cell biology (6). Marshall and colleagues present a complementary view, focusing on the different mechanisms of activation of PI3K in B cells, and the roles of the different phosphatases as well as individual role for additional PIP_3_ effectors such as Bam32, Tapp1, and Tapp2 (7). Puri and Gold pick up on this theme and further explore the clinical utility of blocking PI3K activity in B cells, both in the context of autoimmune diseases, but also by summarizing the early clinical trials that led to accelerated approval of Idelalisib for the treatment of B cell lymphomas (8). Bhatt and Damania consider the roles of PI3K and mTOR in the transformation of B cells by Kaposi sarcoma viruses and how PI3K and/or mTOR inhibitors should be considered for the treatment of virally induced lymphomas (9).

Lewings and colleagues consider how PI3K signaling strength affects T cell differentiation events. Their data show that high levels of PI3K signaling can antagonize the differentiation of Treg in favor of the differentiation of effector T cells (10). Okkenhaug and colleagues present a somewhat different perspective, reviewing evidence that low PI3K activity is also detrimental to Treg function and attempt to reconcile some of the different conclusions regarding the role of PI3K in Treg function (11). Newton and Turka consider the role of the PIP_3_ phosphatase Pten in T cells and how unrestrained PI3K signaling can lead to T cell transformation (12). They further discuss the differential roles of Pten on PI3K signaling and genome stability. Gamper and Powell discuss data that challenge the common assumption that PI3K and mTOR lie on a common pathway, by providing a detailed discussion of similarities but importantly also key differences in the phenotypes observed when either PI3K or mTOR is inhibited in CD4 T cells (13). Similarly, Finlay discusses recent data showing that the PI3K is in fact dispensable for mTOR activation in CD8 T cells and that a Pdk1–mTOR axis regulates T cell metabolism independently of PI3K and Akt (14).

These studies indicate that we need to start considering other signaling pathways regulated by PI3K. Venigalla and Turner summarize recent evidence suggesting a key role for PI3K, often in concert with p38, in regulating mRNA stability and translation (15). Some of these effects may also be mediated by mTOR.

Drugs against PI3K have become a clinical reality. However, not all PI3K isoforms can be targeted as specifically as Idelalisib inhibits PI3Kδ. Blunt and Ward summarized the progress in developing PI3K inhibitors, with particular focus on Idelalisib, but then go on to summarize the roles of the PIP_3_ 5-phosphatases SHIP1 and SHIP2 (16). Moreover, they review the development of allosteric drugs that activate SHIP as an alternative strategy to inhibit PI3K signaling in immune cells.

Altogether, these reviews summarize the remarkable progress in our understanding of how PI3Ks regulate many facets of the adaptive immune response, but also help highlight many unresolved and even controversial areas of research. We are grateful for the considerable efforts that the authors have made to help us compile this ebook for Frontiers in Immunology.

## Conflict of Interest Statement

The authors declare that the research was conducted in the absence of any commercial or financial relationships that could be construed as a potential conflict of interest.
